# Endotherapeutic approach to gastric antral vascular ectasia: Argon plasma coagulation vs. endoscopic band ligation: Randomized clinical trial

**DOI:** 10.1055/a-2868-4601

**Published:** 2026-06-15

**Authors:** Ashok Jhajharia, Shashank Singh, Vivek Pandey, Prachis Ashdhir, Rupesh K. Pokharna, Manoj Verma

**Affiliations:** 1 Gastroenterology 29785 Sawai Man Singh Medical College and HospitalJaipurRajasthanIndia; 2 Community Medicine 482966 Dr Sampurnanand Medical CollegeJodhpurRajasthanIndia

**Keywords:** Endoscopy Upper GI Tract, Non-variceal bleeding, Portal hypertension and variceal bleeding, Ulcers (peptic and other)

## Abstract

**Background and study aims:**

Gastric antral vascular ectasia (GAVE) causes iron-deficiency anemia and gastrointestinal bleeding, sometimes requiring frequent transfusions. Argon plasma coagulation (APC) is effective for treating lesions, but newer methods like endoscopic band ligation (EBL) need to be explored.

**Patients and methods:**

Conducted at a tertiary referral center in India, this single-blinded, prospective, parallel-group randomized study involved 60 patients assigned (1:1) to APC or EBL for endoscopic GAVE obliteration. The primary outcome was clinical success, defined as lesion obliteration and the number of endoscopic sessions required for lesion obliteration. Secondary outcomes included technical success, rebleeding episodes, GAVE recurrence, and hospitalization or transfusion needs. Registered as CTRI Reg. No. CTRI/2023/10/058739.

**Results:**

At 2- and 3-month follow-ups, endoscopic GAVE obliteration was higher in the EBL group (63.3% vs. 30%; *P* = 0.02 and 86.6% vs. 53.3%; *P* = 0.01), but similar at 1- and 6-month follow-ups. Rebleeding episodes were fewer in the EBL group during the initial 3 months (13.3% vs. 40%; *P* = 0.04) but comparable during the subsequent 3 months. Time to rebleeding (weeks) was significantly longer in the EBL group as compared with the APC group (11.6 vs 10.9; *P* = 0.0006). After 6 months, the EBL group had higher hemoglobin levels ( *P* = 0.01) and required fewer transfusions ( *P* = 0.0001). Survival time was comparable between the groups ( *P* = 0.33).

**Conclusions:**

Both EBL and APC effectively treated GAVE lesions and relieved symptoms. Although overall outcomes were similar, EBL required fewer sessions, had less and delayed rebleeding, reduced transfusion needs, and improved hemoglobin levels, indicating higher efficacy in deeper lesion obliteration.

## Introduction

Gastric antral vascular ectasia (GAVE), an uncommon but significant cause of gastrointestinal bleeding, is often associated with chronic blood loss and manifests as iron deficiency anemia 1 . Sometimes, patients may become transfusion-dependent due to recurrent anemia. They may also experience overt gastrointestinal bleeding in the form of intermittent melena or hematemesis 2 .

Population-wide prevalence of GAVE is difficult to determine due to its silent presentation and association with various systemic pathologies 3 . Some studies have found that during routine workup, 3% of patients with advanced liver disease and 2% of those undergoing liver transplantation have GAVE as an additional finding 4 . GAVE is responsible for non-variceal upper gastrointestinal bleeding in 4% of patients with cirrhosis 5 .

Identifying GAVE through endoscopic methods is facilitated by characteristic morphological patterns, such as red hemorrhagic lesions arranged either linearly or in a diffuse punctate (honeycomb) pattern, predominantly located in the gastric antrum but potentially present elsewhere in the stomach 1 . GAVE, also known as "Watermelon stomach," is named for its appearance of parallel red-colored stripes and angiomatous lesions on the antral mucosal folds, resembling watermelon stripes 6 . The linear type is more common in non-cirrhotic patients, with a higher prevalence in older women, whereas the diffuse type is typically observed in cirrhotic patients, predominantly affecting men 7 . Histologically, GAVE is characterized by dilated mucosal capillaries (ectasia) with fibrin thrombi and fibromuscular hyperplasia (spindle cell proliferation) of the lamina propria, without inflammation 8 .

GAVE has been associated with various conditions, including liver cirrhosis, renal failure, bone marrow transplantation, scleroderma, systemic lupus erythematosus, ischemic heart disease, valvular heart disease, hypertension, familial Mediterranean fever, hypothyroidism, hypergastrinemia, diabetes, and acute myeloid leukemia 4 .

Detailed etiopathogenesis of GAVE remains unknown. One major contributing factor is believed to be submucosal fibromuscular hyperplasia and mucosal vessel dilation resulting from altered and dysfunctional antral motility 6 . Among the various therapeutic approaches for symptomatic GAVE, endoscopic techniques are preferred due to the lower risk, ease of application, and minimal side effects 9 . Evidence suggests that argon plasma coagulation (APC), a non-contact thermal coagulation technique using monopolar current via ionized argon gas, is the gold standard therapy for GAVE 10 . Another modality worth mentioning is endoscopic band ligation (EBL), which targets submucosal vascular lesions in GAVE, leading to thrombosis and obliteration 9 . This study aimed to statistically evaluate and compare efficacy, safety, and feasibility of two therapeutic endoscopic approaches—APC and EBL—in ablation of GAVE.

## Patients and methods

**Fig. 1:**
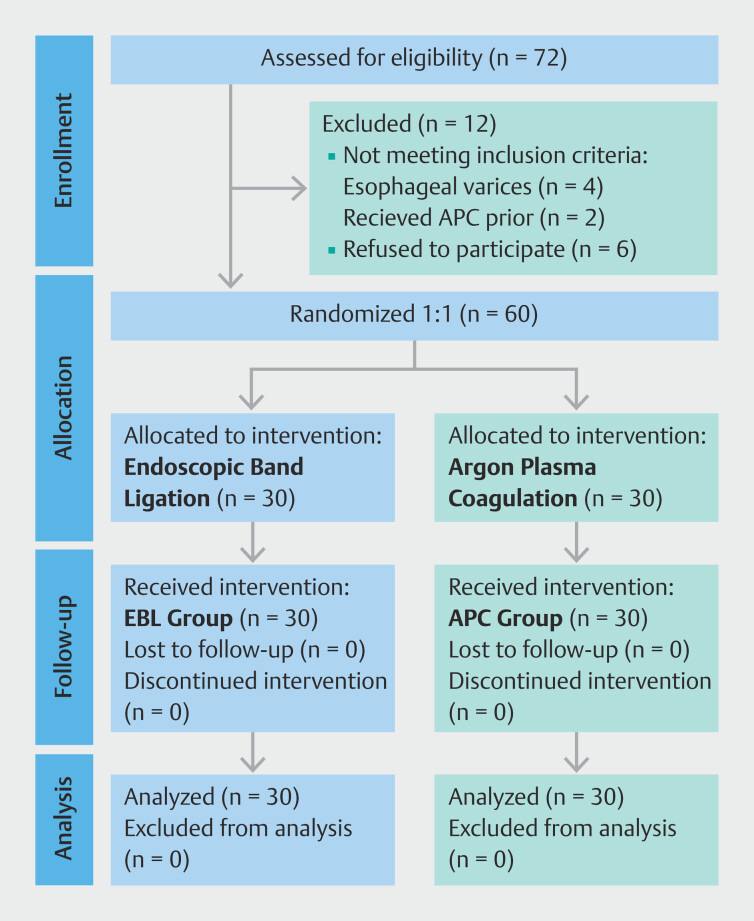
CONSORT flowchart of the study.

This study was performed in a single-blinded, prospective, parallel-group randomized design in the Gastroenterology Department of a leading tertiary referral center in northwest India. Approval for the study was granted by the institutional ethics committee and it was registered with the Clinical Trial Registry of India (ctri.nic.in) under registration number CTRI/2023/10/058739. Patients were randomized in a 1:1 ratio using block randomization based on a computer-generated sequence. Participants were assigned to the group receiving EBL or the group receiving endoscopy guided APC, as illustrated in Fig. 1 . Allocation concealment was maintained using sequentially numbered opaque sealed envelopes, prepared from the random sequence by an individual not involved in patient selection, allocation, or observation. The study complied with principles of the Declaration of Helsinki and all patients provided written informed consent in a comprehensible language.

Sample size was calculated at α error 0.05 with 90% study power. Assuming a difference in mean number of treatment session required to be at least 1 ± 1 as per findings of the study by Abdo M et al. 11 , the minimum required sample size was 42, which was increased to 60 to account for attrition. The null hypothesis considered for the study was that there exists no difference in mean number of treatment sessions required by EBL as compared with endoscopic-guided APC.

**Fig. 2:**
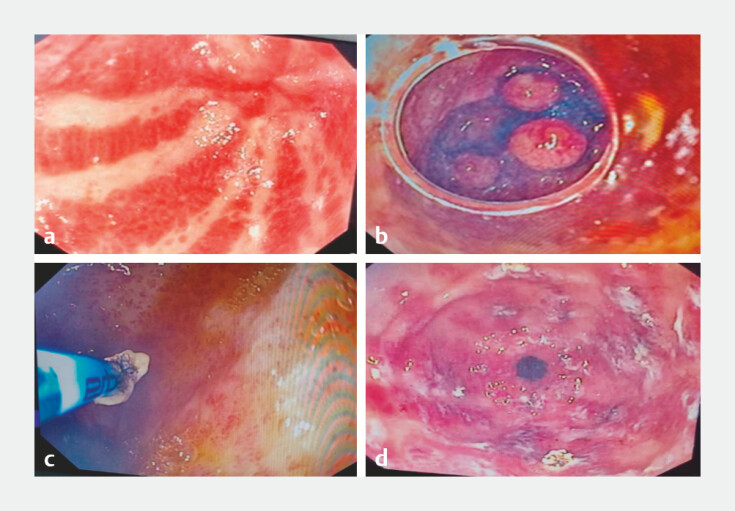
Endoscopic images of gastric antral vascular ectasia lesions.

Patients aged 18 years or older who presented with anemia either having obvious or occult blood loss and who had GAVE confirmed as the bleeding source through upper gastrointestinal endoscopy (UGIE) were included in the study, regardless of GAVE morphology ( Fig. 2 ). Exclusion criteria encompassed patients with bleeding peptic ulcers, bleeding esophageal or gastric varices, bleeding source identified by UGIE other than GAVE, those who had previously received endotherapy for GAVE, patients on antiplatelet or anticoagulant therapy, and individuals with hemorrhagic blood diseases, lymphoproliferative disorders, advanced malignancy or hemorrhagic portal hypertensive gastropathy.

Each patient included in the study underwent comprehensive clinical history, physical examination, biochemistry, and radiological investigations. After obtaining informed consent, eligible patients were followed monthly for 3 months to measure hemoglobin levels and assess GAVE outcomes and any complications from the procedure.

The primary objective of the study was to assess clinical success of the intervention in both groups. Clinical success was determined by obliteration of GAVE lesions, confirmed through upper gastrointestinal endoscopic evaluations conducted monthly for 3 months, and then again at 6 months post-procedure, and the number of endoscopic sessions required for lesion obliteration.

Secondary objectives included technical success, number of sessions needed for complete GAVE obliteration in both groups, number of bands used in the EBL group, adverse events (AEs), long-term endoscopic changes at the GAVE obliteration site, re-bleed episodes at initial 3 months and subsequently in the next 3 months, hemoglobin values at baseline and 6 months postintervention, and number of hospitalizations and blood transfusions in the 6 months before and after the intervention in both groups. Technical success was defined as successful execution of the intended procedure. AEs were classified as mild (minor bleeding, pain), moderate (requiring transfusion or repeat endoscopy), or severe (requiring major intervention, ICU stay, or extended hospitalization) per the American Society for Gastrointestinal Endoscopy lexicon for AEs 12 . Patients who experienced a repeat episode of bleeding (either melena or hematemesis) after the initial intervention were categorized as having a re-bleeding episode.

### Technical aspects of GAVE endotherapy

The procedures were carried out in the Gastroenterology Department, which is equipped with requisite equipment and accessories for therapeutic interventions, as illustrated in Fig. 2 . To ensure consistency and minimize bias, all upper gastrointestinal endoscopies (Olympus GIF-HQ190 with 170 processor) and procedure assessments were conducted by endoscopists (A.J. and P.A.), each with over 15 years of experience in therapeutic endoscopy and teaching. Patients were closely monitored for vital signs with premedications, under the supervision of an anesthetist, throughout the procedure. Midazolam and fentanyl were administered for conscious sedation, if required. All patients underwent pre-procedure assessment and were monitored postintervention while staying in the observation unit. Total procedure time for the index procedure in both groups was measured in minutes, from endoscope insertion to withdrawal after the therapeutic intervention.

### APC technique

In this study, standard APC equipment was utilized, comprising a high-frequency electrosurgical generator (ICC 350; ERBE, Germany), an automatically regulated argon source (APC 300), and a flexible APC probe. The APC probe was a 2.3-mm Teflon-coated catheter with a thermo-resistant ceramic tip, designed to be passed through the endoscope working channel. Electrical power was set at 60 W, with an argon gas flow of 2 L/min. APC was applied to the lesion starting at the pylorus and moving proximally. A foot pedal was used to control application time for coagulation, targeting mucosal spots or lines of GAVE until blanching occurred. Attention was given to avoid direct or prolonged contact to prevent deep tissue necrosis or scarring.

### Band ligation technique

EBL was performed using six-shot over-the-scope band ligation sets with a pneumo-activated suction device. Bands were applied to abnormal GAVE mucosa starting in the distal gastric antrum and moving proximally in a circular manner around the pylorus until the maximal abnormal mucosa affected by GAVE was ligated. Distance between the bands was maintained at approximately 2 to 3 cm, with a maximum of eight bands allowed per session. After each EBL session, all patients received an injectable proton pump inhibitor (PPI), followed by oral PPI (administered every 12 hours) for a minimum of 7 days to promote early mucosal healing.

### Follow-up

Sessions were conducted every month in a periodic manner until complete eradication of GAVE lesions was achieved. In the event of recurrence of or overt or occult upper gastrointestinal bleeding, patients from either group were reevaluated for residual GAVE lesions. Hemoglobin levels and other biochemical parameters were assessed monthly. There were no restrictions on blood transfusions, iron supplementation, or hospitalizations if clinically indicated. To document endoscopically proven recurrence of GAVE lesions during follow-up, a minimum period of 6 months from the index session was considered.

### Statistical analysis

Categorical variables were described using frequencies and percentages, whereas quantitative variables were summarized by their mean and standard deviation along with confidence intervals (CIs) or median and interquartile range (IQR). Categorical variables were analyzed using the Chi-square test or Fisher's exact test and the Mann-Whitney U test was employed to compare quantitative variables.

Over the follow-up period, survival time and duration until rebleed incidence between the two study groups were compared using Kaplan-Meier estimates and log-rank analysis. Statistical significance was defined as *P* < 0.05 (two-sided), with all analyses conducted using IBM SPSS Statistics Version 27.

## Results

The study included a total of 60 patients, with 30 assigned to the EBL group and 30 to the APC group. Baseline sociodemographic, biochemical, and clinical data showed no significant differences between the two groups, as detailed in Table 1 .

**Table:** **Table 1** Sociodemographic and clinical characteristics of study groups.

	EBL group (n = 30)	APC group (n = 30)	*P* value*
Age, years; median (IQR)	43.5 (38.6–48.4)	43.9 (39.0–48.8)	0.944 ^†^
Gender, n (%)			0.561 ^‡^
Male	20 (66.6%)	23 (76.6%)	
Female	10 (33.3%)	7 (23.3%)	
Hematemesis, n(%)	2 (6.6%)	1 (3.3%)	1.00 ^‡^
Malena, n(%)	14 (46.6%)	17 (56.6%)	0.605 ^‡^
GAVE pattern			0.398 ^‡^
Classic (watermelon)	23 (76.6%)	19 (63.3%)	
Punctuate (diffuse)	07 (23.3%)	11 (36.6%)	
Etiology of GAVE			0.529 ^§^
CLD	14 (46.6%)	17 (56.6%)	
CKD	5 (16.6%)	6 (20.0%)	
Scleroderma	2 (6.6%)	0	
Others	9 (30.0%)	7 (23.3%)	
Etiology of CLD			0.484 ^§^
Ethanol	9 (64.2%)	13 (76.4%)	
HBV	1 (7.1%)	2 (11.7%)	
HCV	0	1 (5.8%)	
Others	4 (28.5%)	1 (5.8%)	
Hemoglobin, g/dL; median (IQR)	6.7 (5.5–8.0)	7.0 (6.1–7.9)	0.312 ^†^
Platelets, ×1000/µL; median (IQR)	70.1 (53.1–87.1)	73.5 (57.8–89.2)	0.453 ^†^
INR; median (IQR)	1.79 (1.36–2.21)	2.02 (1.54–2.50)	0.183 ^†^
WBC, ×1000/µL; median (IQR)	7.53 (6.43–8.63)	7.83 (6.84–8.81)	0.441 ^†^
Total bilirubin, mg/dL; median (IQR)	2.73 (1.76–3.70)	2.44 (1.60–3.28)	0.332 ^†^
ALT, (IU/L); median (IQR)	92.6 (51.2–134.0)	98.9 (39.2–158.6)	0.645 ^†^
AST, (IU/L); Median (IQR)	81.1 (55.7–106.6)	87.1 (42.6–131.5)	0.681 ^†^
Serum albumin, (mg/dL); median (IQR)	2.54 (1.93–3.15)	2.22 (1.73–2.71)	0.152 ^†^
^*^ Significance level = 0.05. ^†^ Mann-whitney U test. ^‡^ Fischer’s exact test. ^§^ Chi-square test. ALT, alanine aminotransferase; APC, argon plasma coagulation; AST, aspartate aminotransferase; CKD, chronic kidney disease; CLD, chronic liver disease; EBL, endoscopic band ligation; HBV, hepatitis B virus; GAVE, gastric antral vascular ectasia; HCV, hepatitis C virus; INR, international normalized ratio; IQR, interquartile range; WBC, white blood cell.			

**Fig. 3:**
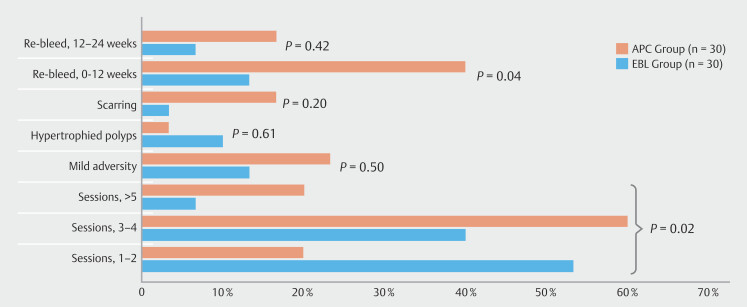
Bar chart of outcome parameters among the study groups.

The primary outcome was complete obliteration of endoscopically visible GAVE lesions following intervention, as shown in Table 2 and the number of sessions required to eliminate GAVE lesions which was further categorized into three groups: one to two sessions, three to four sessions, and five or more sessions, as outlined in Table 3 and Fig. 3 . Patients were assessed monthly over a 3-month period after the initial intervention. By 1 month, complete obliteration was achieved in 16.6% of the EBL group and 20.0% of the APC group ( *P* = 1.00). The remaining 83.3% in the EBL group and 80.0% in the APC group, who only had partial obliteration, underwent repeat sessions of the same intervention as initially assigned ( Table 2 ). At 2 and 3 months, the EBL group showed higher complete obliteration than the APC group ( *P* = 0.02 and 0.01, respectively).

**Table:** **Table 2** Primary outcome: Obliteration of endoscopic visible GAVE.

	EBL group (n = 30)	APC group (n = 30)	*P* value*
At 1 month			1.00 ^†^
Complete obliteration, n (%)	5 (16.6%)	6 (20.0%)	
Partial obliteration, n (%)	25 (83.3%)	24 (80.0%)	
At 2 months			**0.019** ^†^
Complete obliteration, n (%)	19 (63.3%)	9 (30.0%)	
Partial obliteration, n (%)	11 (36.6%)	21 (70.0%)	
At 3 months			**0.010** ^†^
Complete obliteration, n (%)	26 (86.6%)	16 (53.3%)	
Partial obliteration, n (%)	4 (13.3%)	14 (46.6%)	
At 6 months			0.299 ^†^
Complete obliteration, n (%)	27 (90.0%)	23 (76.6%)	
Partial obliteration, n (%)	3 (10.0%)	7 (23.3%)	
Adverse events			0.506 ^†^
Minor, n (%)	4 (13.3%)	7 (23.3%)	
Major, n (%)	–	–	
Long-term changes (6 months)			
Hypertrophied polyps, n (%)	3 (10.0%)	1 (3.3%)	0.612 ^†^
Scarring/fibrosis, n (%)	1 (3.3%)	5 (16.6%)	0.194 ^†^
Ulcer, n (%)	–	–	–
*Significance level = 0.05. ^†^ Fischer’s exact test. APC, argon plasma coagulation; EBL, endoscopic band ligation; GAVE, gastric antral vascular ectasia.			

**Table:** **Table 3** Intervention and primary outcome among the study groups.

	EBL group (n = 30)	APC group (n = 30)	*P* value*
Sessions, n (%)			** 0.020 ^†^**
1–2	16 (53.3%)	6 (20.0%)	
3–4	12 (40.0%)	18 (60.0%)	
≥ 5	2 (6.6%)	6 (20.0%)	
No. of bands used, n (%)		–	–
< 6	14 (46.6%)		
7–12	13 (43.3%)		
≥ 13	3 (10.0%)		
Duration of index procedure, minutes; median (IQR)	15.2 [15.0–15.5 ]	25.8 [25.4–26.2]	** < 0.05 ^‡^**
^*^ Significance level = 0.05. ^†^ Chi-square test. ^‡^ Mann-Whitney U test. APC, argon plasma coagulation; EBL, endoscopic band ligation; IQR, interquartile range.			

To assess persistence of obliteration, patients underwent endoscopic evaluation 6 months after the initial intervention. At 6 months, complete obliteration was observed in 90.0% (EBL group) vs. 76.6% in the APC group ( *P* = 0.30). No serious AEs were reported in either group during or after the intervention. However, mild AEs were noted in 13.3% of patients in the EBL group and 23.3% in the APC group. Abdominal pain occurred in 13.3% of the EBL group and 16.6% of the APC group, alleviated with oral analgesics. Of the patients in the APC group, 6.6% developed fever shortly after the intervention, which resolved with antipyretics.

During 6-month follow-up, patients were also evaluated for post-procedure changes at the GAVE site. Hypertrophied polyps were observed in 10.0% of patients in the EBL group, compared with only one patient (3.3%) in the APC group ( *P* = 0.61). Fibrotic sequelae in the form of scarring were observed in 3.3% of the EBL group, compared with 16.6% of the APC group ( *P* = 0.20), as shown in Table 2 . Persistent ulceration was not observed at the intervention site in either group during 6-month follow-up.

All patients were monitored for upper gastrointestinal bleeding recurrence (hematemesis or melena) over 6-month follow-up, split into two phases. In the initial 3 months (0–12 weeks), 13.3% of the EBL group had symptoms compared with 40.0% of the APC group ( *P* = 0.04). In the subsequent 3 months (12–24), recurrence occurred in 6.6% of EBL patients and 16.6% of APC patients ( *P* = 0.42) ( Table 4 ). Relative risk of rebleeding with EBL vs. APC was 0.35, indicating a 65% relative risk reduction.

**Table:** **Table 4** Comparative analysis of secondary outcomes among study groups.

	EBL group (n = 30)	APC group (n = 30)	*P* value*
Rebleeding episodes (hematemesis/malena)			
Initial 3 months (0–12 weeks), n (%)	4 (13.3%)	12 (40.0%)	** 0.039 ^†^**
Subsequent 3 months (12–24 weeks), n (%)	2 (6.6%)	5 (16.6%)	0.423 ^†^
Duration till re-bleed events, mean ± SD, weeks [95% CI]	11.6 ± 7.0 [6.0–17.2]	10.9 ± 5.2 [8.5–13.3]	** 0.0006 ^‡^**
Baseline			
Hemoglobin (g/dL); median (IQR)	6.7 (5.5–8.0)	7.0 (6.1–7.9)	0.312 ^‡^
Hospitalization (previous 6 months); median (IQR)	2 (1.6–2.4)	2 (1.5–2.5)	0.583 ^‡^
Blood transfusion (previous 6 months); Median (IQR)	3 (2.4–3.6)	2 (1.1–2.9)	0.170 ^‡^
Follow up			
Hemoglobin (g/dL), at 6 months; median (IQR)	10.1 (9.3–10.9)	9.1 (8.0–10.2)	**0.012** ^‡^
Hospitalization in 6 months; median (IQR)	1 (0.6–1.4)	1 (0.6–1.4)	0.849 ^‡^
Blood transfusion in 6 months; median (IQR)	1 (0.6–1.4)	2 (1.4–2.6)	**0.00016** ^‡^
Change (Δ) in hemoglobin, (g/dL); median (IQR)	3.4 (2.0–4.8)	2.0 (0.5–3.6)	**0.019** ^‡^
Change (Δ) in number of hospitalizations; median (IQR)	1.0 (0.4–1.6)	0.9 (0.3–1.5)	0.787 ^‡^
Change (Δ) in number of blood transfusions; median (IQR)	2.0 (1.3–2.7)	0.83 (-0.24–1.90)	**0.002** ^‡^
Death during follow-up, n (%)	2 (6.6%)	4 (13.3%)	0.670 **^†^**
^*^ Significance level = 0.05. ^†^ Fischer’s exact test. ^‡^ Mann-Whitney U test. APC, argon plasma coagulation; CI, confidence interval; EBL, endoscopic band ligation; IQR, interquartile range; SD, standard deviation.			

At baseline, median hemoglobin levels, median hospitalizations, and median packed red blood cell units transfused in the past 6 months were similar between the EBL and APC groups. During 6-month follow-up, median hemoglobin was 10.1 g/dL in the EBL group vs. 9.1 g/dL in the APC group ( *P* = 0.01). Median number of hospitalizations was one in both groups ( *P* = 0.85). Median PRBC units transfused was one in the EBL group, significantly lower than two in the APC group ( *P* = 0.0002). Median hemoglobin increase from baseline to 6 months was 3.4 g/dL in the EBL group vs. 2.02 g/dL in the APC group ( *P* = 0.02). Median change in hospitalizations over 6-month follow-up was similar between groups ( *P* = 0.79). Median change in PRBC units transfused was two for EBL vs. 0.83 for APC ( *P* = 0.002).

Within-group analysis showed significant differences in the EBL group between baseline/past 6-month values and postintervention/6-month follow-up for median hemoglobin, median hospitalizations, and PRBC units transfused ( *P* < 0.00001, for all). Similarly, the APC group showed significant changes, with *P* < 0.0001 for median hemoglobin and hospitalizations, and *P* = 0.023 for PRBC units transfused ( Table 5 ).

**Table:** **Table 5** Intra-group analysis of secondary outcomes in both study groups.

	EBL group (n = 30)			APC group (n = 30)		
	Pretreatment (baseline/previous 6 months)	Post-treatment (6 months)	*P* value ^*†^	Pretreatment (baseline/previous 6 months)	Post-treatment (6 months)	*P* value ^*†^
Hemoglobin	6.7 (5.5–8.0)	10.1 (9.3–10.9)	**< 0.001**	7.0 (6.1–7.9)	9.1 (8.0–10.2)	**< 0 .001**
Hospitalization	2 (1.6–2.4)	1 (0.6–1.4)	**< 0.001**	2 (1.5–2.5)	1 (0.6–1.4)	**< 0.001**
Transfusion	3 (2.4–3.6)	1 (0.6–1.4)	**<0 .001**	2 (1.1–2.9)	2 (1.4–2.6)	**0.0226**
^*^ Significance level = 0.05. ^†^ Wilcoxon signed rank test. Data presented in median (IQR). APC, argon plasma coagulation; EBL, endoscopic band ligation; IQR, interquartile range.						

**Fig. 4:**
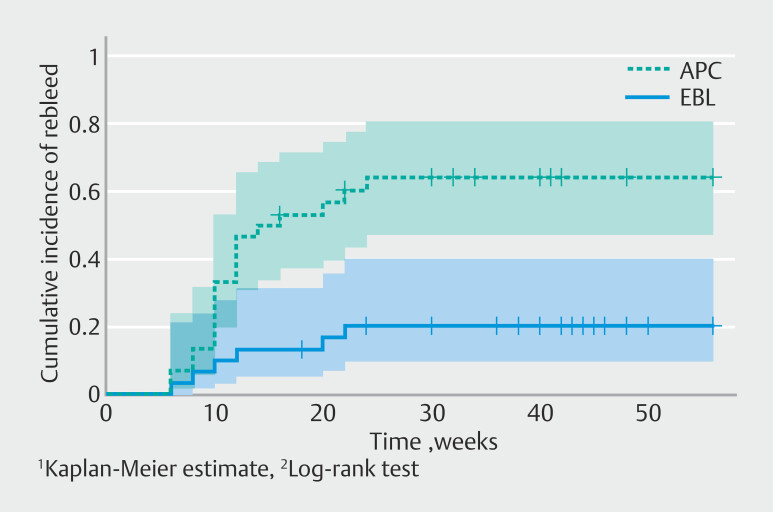
Cumulative incidence of re-bleeding curve.

Kaplan-Meier analysis compared cumulative rebleeding incidence in symptomatic upper gastrointestinal bleeding patients between the two groups ( Fig. 4 ). Cumulative incidence of rebleeding over 24 weeks was 20.0% (95% CI 5.7%-34.3%) in the EBL group and 55.5% (95% CI 40.2%-70.8%) in the APC group. The EBL group had a significantly lower rebleeding incidence than the APC group (log-rank, *P* = 0.0006). Reinterventions were performed according to initial group allocation, at monthly intervals.

**Fig. 5:**
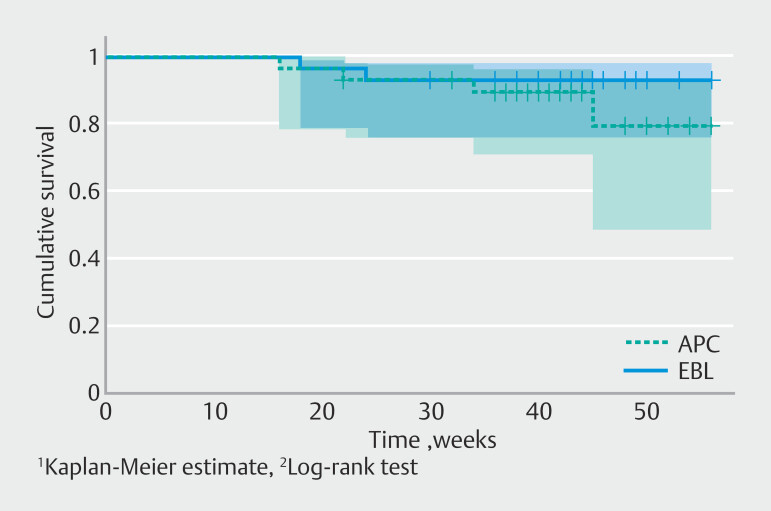
Cumulative survival plot.

Patients were followed for a minimum period of 6 months to assess disease-related mortality. Cumulative incidence of mortality was 6.6% (95% CI 0%-15.4%) in the EBL group and 13.3% (95% CI 3.5%-23.1%) in the APC group, with no significant difference in Kaplan-Meier survival between groups (log-rank *P* = 0.33) ( Fig. 5 ).

## Discussion

In this comparative study, we aimed to evaluate efficacy of endo-therapeutic approaches for eradication of GAVE lesions and resolution of symptoms by comparing two methods, EBL and APC, in a randomized manner. GAVE can present symptomatically as anemia or as manifestations of upper gastrointestinal bleeding. In this study, we observed that all patients had anemia, with 46.6% in the EBL group and 56.6% in the APC group experiencing melena, consistent with previous studies 1 2 11 . This study also explored the varied etiological spectrum of GAVE lesions and found minimal correlation with severity and causation, including chronic liver disease, chronic kidney disease, diabetes, or other conditions, as supported by previous research 4 . The objective was to statistically compare and evaluate outcomes and success of APC and EBL in controlling bleeding, eradicating lesions, reducing need for blood transfusions, and overall morbidity among patients with bleeding GAVE, regardless of associated systemic diseases in the northwestern region of the Indian subcontinent.

APC has eradication efficacy of 80% to 100% on GAVE lesions, and after two to three sessions on average, need for blood transfusions to maintain hemoglobin levels decreases in 50% to 80% of patients 10 . However, its long-term efficacy for eradication of GAVE and exclusivity remains in question 9 . Settings for APC, including power output and contact time, have shown varied efficacy in research studies, highlighting need for a standardized, uniform approach and definition 13 . Another promising and comparable alternative for treating deeper mucosal and submucosal vascular lesions in GAVE patients is EBL, which can treat more than the superficial mucosal lesions ablated by APC 9 . This study provided a head-to-head comparison of APC and EBL with the primary outcome being GAVE lesion obliteration and number of endoscopic sessions required for obliteration. The EBL group required statistically fewer sessions to achieve maximal obliteration compared with the APC group, consistent with results from a meta-analysis by Hirsch BS et al., which favored EBL for endoscopic eradication 14 . In the EBL group, the number of bands used per patient varied widely, similar to findings from a study by Elhendawy et al., which suggested using up to 12 bands 15 . In this study, mean duration (minutes) of the index intervention was significantly shorter in the EBL group compared with the APC group ( *P* < 0.05), likely due to greater area coverage by bands and the longer time required for focal localization of the APC probe on target lesions.

In this study, EBL-induced obliteration of endoscopic GAVE lesions showed a significantly higher proportion at the second and third month of follow-up after the initial intervention, although the APC group performed comparably after 1 and 6 months of follow-up. Similar findings were observed in the study conducted by Nassar et al 16 . Procedure-related AEs in this study were all mild and comparable between the two groups, not requiring prolonged stays or additional interventions, similar to what was observed in the study conducted by Elhendawy et al 15 . No patients in the EBL group developed post-EBL ulcers, likely due to fewer bands used, antacid medication, and continued treatment of the underlying causable site polyps near the antrum were observed at 6-month follow-up in 10.0% of patients in this study, a slightly lower observation than the findings of Abdo et al 11 . Scarring at APC sites was observed in 16.6% of patients, a common post-procedure finding.

As part of the secondary outcomes, rebleeding episodes, an indicator of therapeutic efficacy of endoscopic modalities, were assessed at two intervals: the initial 3 months (0–12 weeks) and the subsequent 3 months (12–24 weeks). The APC group became more symptomatic in terms of upper gastrointestinal bleeding (hematemesis/melena) compared with the EBL group during the initial 3 months. However, during the subsequent 3 months, rebleeding incidences were comparable between the two groups. A similar observation was made in favor of EBL in a meta-analysis conducted on 116 patients 14 . This could be due to the deeper submucosal-targeted vascular thrombosis and subsequent eradication of GAVE lesions, facilitated by the suction and contraction force used in EBL with the aid of a pneumatic endoscopic device. Following the initial procedure (done at week 0), mean duration until the first rebleeding incident was significantly longer in the EBL group compared with the APC group, as shown by log-rank analysis. In a comparable study, a significantly higher recurrence of bleeding was observed in the APC group 17 .

Within-group analysis unequivocally supported long-term therapeutic efficacy of both approaches, EBL and APC, in improving hemoglobin levels, decreasing need for transfusions or hospitalizations. This study aimed to find the optimum approach for a better and more efficacious outcome for symptomatic GAVE patients. The aforementioned parameters were comparable in both groups at baseline. After 6 months of follow-up, mean hemoglobin values achieved were significantly higher in the EBL group compared with the APC group. The EBL group also showed a decreased need for blood transfusions over the follow-up period. Mean change in hemoglobin values and blood units transfused also significantly favored the EBL group compared with the APC group when comparing values from baseline or the previous 6 months until the study endpoint. Previous meta-analyses also suggested that EBL is associated with better hemoglobin improvement, lesser transfusion requirements, shorter hospital stays, fewer rebleeding occurrences, and fewer treatment sessions 18 .

Mortality during the follow-up period, although not a predetermined outcome due to the wide spectrum of underlying systemic diseases for which symptomatic GAVE is only a point feature, was comparable between the groups. Moreover, median follow-up duration of patients in both groups, serving as an auxiliary measure of median survival, was similar in the log-rank test between the two groups.

Considering practicality, EBL offers higher availability in low-resource settings, is more cost-effective, requires less expertise, and thus, has uniform applicability 19 . In a study of patients with bleeding GAVE, Ghaffar and Abd El Magaud concluded that both APC and EBL are efficacious and safe approaches, with the latter being more effective and time-saving 17 . EBL therapy for GAVE is still evolving because there is no clear consensus on ideal usage, number of sessions needed for ablation, number of bands per session, or interval between sessions. Not all studies unanimously favored EBL; for instance, a study by Fabian et al. showed no significant impact on hemoglobin levels or transfusion needs 20 . Using a combined approach, APC alternating with EBL has proven more efficacious than EBL alone, as demonstrated in a study by Abdo et al. for treating bleeding GAVE, resulting in a reduced incidence of band-related ulcers or polyps 11 . Newer therapies, such as radiofrequency ablation (RFA), have been found effective in GAVE eradication, although comparative data remain limited 18 .

The primary limitation of this study is the small sample size, restricting generalizability. Patients were followed for 6 months to assess symptom persistence or GAVE lesion recurrence. Longer follow-up could provide more comprehensive analysis. Despite advantages of each approach, generalizability remains uncertain because the method should fit the specific case. This study uniquely includes patients with various etiologies, not limited to cirrhosis, unlike most prior studies.

## Conclusions

This study compared EBL and APC for treating GAVE. Both methods effectively obliterated GAVE lesions, were successfully executed, mitigated bleeding, and reduced blood transfusion needs, with EBL showing a higher rate of lesion obliteration, less bleed recurrence, and longer time until rebleeding. Inclusion in tte study of a diverse patient population adds to the existing literature, suggesting that EBL may be more efficient and widely available in resource-limited settings. The study also highlights the efficacy of APC and EBL as standalone endoscopic procedures to eradicate and minimize bleeding from GAVE lesions.
